# A novel hypoxia-dependent 2-nitroimidazole KIN-841 inhibits tumour-specific angiogenesis by blocking production of angiogenic factors

**DOI:** 10.1038/sj.bjc.6600667

**Published:** 2003-01-28

**Authors:** M Shimamura, H Nagasawa, H Ashino, Y Yamamoto, T Hazato, Y Uto, H Hori, S Inayama

**Affiliations:** 1 Medical R&D Center, The Tokyo Metropolitan Institute of Medical Science, 3-18-22 Honkomagome, Bunkyo-ku, Tokyo 113-8613, Japan; 2 Department of Biological Science and Technology, Faculty of Engineering, The University of Tokushima, Minamijosanjimacho Tokushima 770-8506, Japan; 3 Department of Molecular Biology, The Tokyo Metropolitan Institute of Medical Science, 3-18-22 Honkomagome, Bunkyo-ku, Tokyo 113-8613, Japan; 4 Pharmaceutical Institute, School of Medicine, Keio University, 35 Sninanomachi, Shinjuku-ku, Tokyo 160-8582, Japan; 5 Institute of Oriental Medical Sciences, 2-6-3 Ebisunishi, Shibuya-ku, Tokyo 155-0021, Japan

**Keywords:** angiogenesis, endothelial cells, hypoxia, angiogenic factor, nitroimidazole

## Abstract

Tumour angiogenesis is initiated by angiogenic factors that are produced in large amounts by hypoxic tumour cells. The inhibition of this step may lead to tumour-specific antiangiogenesis because normal tissues are not usually hypoxic. On the other hand, blocking a biological function of endothelial cells is known to result in angiogenic inhibition. To produce a tumour-specific and powerful antiangiogenesis, we determined whether potent angiogenic inhibition could be achieved by inhibiting the production of angiogenic factors by hypoxic tumour cells and simultaneously blocking certain angiogenic steps in endothelial cells under normoxia. We focused on the 2-nitroimidazole moiety, which is easily incorporated into hypoxic cells and exhibits its cytotoxicity as hypoxic cytotoxin. We designed and synthesised 2-nitroimidazole derivatives designated as KIN compounds, and investigated their antiangiogenic activities under normoxia using a chick embryo chorioallantoic membrane. KIN-841 (2-nitroimidazole 1-acetylhydroxamate) showed a potent angiogenic inhibition in a dose-dependent manner. This compound inhibited the proliferation of bovine pulmonary arterial endothelial (BPAE) cells more strongly than that of tumour cells, such as Lewis lung carcinoma (3LL) cells, under normoxia. The inhibition of cell proliferation by KIN-841 under hypoxia increased about five-fold compared to that under normoxia. Moreover, under hypoxia, KIN-841 significantly decreased the excessive production of vascular endothelial cell growth factors induced by 3LL cells as determined by tritium-labelled thymidine ([^3^H]thymidine) incorporation into BPAE cells and by ELISA. Intraperitoneal administration of KIN-841 suppressed 3LL-cell-induced *in vivo* angiogenesis in the mouse dorsal air sac system. These results indicate that the regulation of the production of angiogenic factors by hypoxic tumour cells is a useful target for tumour-specific angiogenesis inhibition, and that KIN-841, which causes simultaneous direct inhibition of endothelial cell function and production of angiogenic factors by hypoxic tumour cells, is a very potent inhibitor of tumour-specific angiogenesis. Thus, the potential for clinical use of KIN-841 as an antitumour drug is very high.

Angiogenesis is the formation of new blood vessels, and is essential for tissue development, regeneration and remodelling (Folkman and Shing, 1992). Angiogenesis also plays an important role in many pathological processes, such as growth and metastasis of solid tumour, diabetic retinopathy, rheumatoid arthritis and psoriasis. The inhibition of angiogenesis results in a suppression of these diseases.

The initiation of angiogenesis is associated with some changes in the environment affecting the equilibrium between angiogenic factors and inhibitors. Hypoxia is considered as the leading cause of angiogenesis. In solid tumours, hypoxia exists between the aerobic region near the capillary blood vessels and the anaerobic region where necrotic cells are present. Hypoxia is a common feature of human and animal tumours (Moulder and Rockwell, 1984; Urtasun *et al*. 1986). Recently, the expression of vascular endothelial growth factor (VEGF) was shown to increase significantly in human gliomas under hypoxic conditions (Shweiki *et al*. 1992). In human melanoma cells xenografted to nude mice, the mRNAs coding for VEGFs were significantly elevated under hypoxia, which resulted in tumour angiogenesis (Potgens *et al*. 1995). Hypoxia thus upregulates the expression of VEGF in tumour cells, which subsequently leads to angiogenesis. Therefore, the downregulation of the production of angiogenic factors by hypoxic tumour cells is a potential therapeutic method of inhibiting tumour-specific angiogenesis. On the other hand, inhibition of certain steps in the angiogenic process in endothelial cells, such as proliferation, migration and tube formation, undoubtedly results in antiangiogenesis. Simultaneously reducing the levels of angiogenic factors in hypoxic tumour cells and blocking the biological function of endothelial cells under normoxia can induce much more potent angiogenic inhibition in solid tumours.

Hypoxic tumour cells represent a significant problem in radiation therapy and chemotherapy since they are resistant to X-ray and antitumour drugs. The use of a radiosensitiser with an affinity for hypoxic tumour cells and a probe for the detection and quantitation of hypoxia regions in tumours is important for developing appropriate therapeutic modalities against hypoxic tumour cells. Nitroimidazole derivatives have been studied extensively for use as radiosensitisers (Sheldon *et al*. 1974; Brown, 1975) and molecular markers of hypoxic regions in solid tumours (Walton and Workman, 1987; Aboagye *et al*. 1995). 2-Nitroimidazole derivatives, such as misonidazole and etanidazole, have been reported to have potent radiosensitising ability (Sheldon *et al*. 1974; Brown *et al*. 1981), and have been clinically studied regarding their application in the radiotherapy of patients with malignant tumours (Chang *et al*. 1998; Grigsby *et al*. 1999). The efficiencies of nitroimidazoles as hypoxic radiosensitisers depend on the selective and irreversible binding to the reactive metabolite of the nitro group having one-electron reduction potentials to target molecules in hypoxic tumour cells (Adams *et al*. 1979). Although nitroimidazole derivatives have been developed as radiosensitisers, they also show preferential toxicity to hypoxic cells as hypoxic cytotoxins (Stratford and Stephens, 1989; Papadopoulou *et al*. 1996). The cytotoxicity of nitroheterocycles such as misonidazole and etanidazole toward hypoxic cells is a result of abstraction of hydrogen from target molecules by free radicals formed in the reduction of the nitro group. The efficiencies of both radiosensitising activity and cytotoxicity toward hypoxic tumour cells depend on the nitro group of 2-nitroimidazoles (Varghese and Whitmore, 1980; Bump *et al*. 1983). We designed and synthesised 2-nitroimidazole derivatives termed KIN compounds, and showed that KIN-802 and KIN-806 are potent hypoxic cell radiosensitisers (Hori *et al*. 1989; Nagasawa *et al*. 1992).

In this study, we achieved potent inhibition of tumour-specific angiogenesis by inhibiting certain angiogenic steps under both normoxia and hypoxia using the novel hypoxic-dependent 2-nitroimidazole KIN-841.

## Materials and Methods

### Compounds

All the KIN compounds tested (namely KIN-801, KIN-804, KIN-806, KIN-808, KIN-811, KIN-831 and KIN-841), which were 2-nitroimidazole derivatives with a moiety of –(CH_2_)_*n*_CONHR in the side chain, were synthesised and their structures were identified based on the physical and spectral data as described previously (Hori *et al*. 1989; Nagasawa *et al*. 1992). Misonidazole, etanidazole and 2-nitroimidazole were obtained from Nippon Roche (Tokyo, Japan). The anti-mouse VEGF polyclonal antibody and ELISA kit for the determination of mouse VEGFs were purchased from R&D Systems Inc. (Minneapolis, MN, USA).

### Cells

Bovine pulmonary arterial endothelial (BPAE) cells were obtained from Dr T Takano (Jintan Terumo, Kanagawa, Japan). Lewis lung carcinoma and cultured Lewis lung carcinoma (3LL) cells were from Dr T Sakurai (Keio University, Tokyo, Japan). 3LL cells were grown in Eagle's medium supplemented with 10% fetal calf serum (FCS) and BPAE cells were maintained in Dulbecco's MEM containing 10% FCS.

### Chick embryo chorioallantoic membrane assay

Angiogenic activity was assayed using a chick embryo chorio-allantoic membrane (CAM) as described previously (Shimamura *et al*. 2001). Using a 4-day-old chick embryo (Ohmiya Kakin Laboratories, Ohmiya, Japan) in a shell, 10 *μ*l of sample mixed in 1% methyl cellulose/0.9% NaCl was applied to the ring on the surface of the CAM. After 48 h of exposure at 37°C, a fat emulsion (Mitsubishi Pharma Corporation, Osaka, Japan) was injected into the CAM to visualise clearly the blood vessels. Each experimental group included six eggs, and experiments were repeated five times. Angiogenic inhibition was indicated by the presence of a 3-mm-diameter avascular zone around the ring (Oikawa and Shimamura, 1996; Yamaji *et al*. 1999). Inhibition of angiogenesis (%) means (number of eggs showing at least 3 mm zone of inhibition)/(number of eggs used in each experimental group)×100.

### Cell proliferation under normoxic condition

Cultured BPAE cells and 3LL cells were seeded on 24-multiwell plates at 5×10^4^ cells per well in a medium containing 10% FCS. After 24 h of incubation, the medium was discarded and various concentrations of KIN-841 in fresh medium containing 3% FCS were added. On day 3, the cells were trypsinised and counted using a Coulter counter. Each experimental group included five wells, and experiments were repeated three times.

### Cell proliferation under hypoxic condition

3LL cells were seeded on Petri dishes at 5×10^4^ cells ml^−1^ in a medium containing 10% FCS. After 24 h of incubation, the medium was discarded and the cells were washed with PBS. Fresh medium containing 5% FCS and various concentrations of KIN-841 were added into the Petri dishes, which were incubated for 48 h under hypoxic conditions. For incubation under hypoxia, the cells in the Petri dishes were arranged in acrylic Lucite chambers to which a gas mixture (95% N_2_–5% CO_2_) was infused at a flow rate of 1 l min^−1^ (Iizuka *et al*. 1994). Control endothelial cells were maintained under normoxia. The cells in the Petri dishes were trypsinised and counted using a Coulter counter. Each experimental group included five wells, and experiments were repeated three times.

### Angiogenic factor production

3LL cells were cultured in the medium containing 10% FCS. At confluence, the medium was changed to a serum-free one with or without various concentrations of KIN-841. After incubation for 24 h under normoxia or hypoxia, the conditioned medium (CM) was collected for the assay of [^3^H]thymidine incorporation in BPAE cells and for ELISA. For [^3^H]thymidine incorporation, 3×10^4^ BPAE cells were seeded on 96-well plates. After 24 h the medium was discarded, and serum-free medium and the five-fold diluted CM obtained from 3LL cells treated with or without KIN-841 were added to the wells. On the other hand, for the assay of the direct effect of KIN-841 on BPAE cells, an appropriately diluted KIN-841-free CM from normoxic or hypoxic 3LL cells was mixed with various concentrations of KIN-841 and added to the BPAE cell-seeded wells. For the assay with anti-mouse VEGF-neutralizing antibody, a diluted CM from hypoxic or normoxic 3LL cells was mixed with anti-mouse VEGF antibody (1 : 1000) for the 24-h incubation at 4°C, and then added to the BPAE cell-seeded wells for [^3^H]thymidine incorporation assay. The treated BPAE cells were incubated for 8 h, and then 37 kBq per well of thymidine-[methyl-^3^H] (NEN, Wilmington, DE, USA) was added. After 16 h of incubation, the medium was discarded and the cells were washed with PBS and lysed in 4% Na_2_CO_3_/2N NaOH. The radioactivity in 20 *μ*l of the cell lysate was determined using a scintillation counter. The presence of VEGF in the CM from 3LL cells was evaluated using ELISA. Each experimental group included three wells, and experiments were repeated three times.

### Dorsal air sac assay

Millipore chambers covered with filters (0.45-mm pore size) were filled with either saline or 4×10^6^ 3LL cells, and were implanted s.c. in dorsal air sacs created surgically by injection of an appropriate volume of air to 7-week-old female ICR mice (Charles River Co. Ltd, Yokohama, Japan). The mice were administered with KIN-841 (200 mg kg^–1^ day^−1^) or saline intraperitoneally for 4 days between days 1 and 6. On day 7, mice were killed under diethyl ether and the skin was carefully removed. Each experimental group included six mice, and experiments were repeated twice. Angiogenesis was graded using four angiogenesis indices of 1, 2, 3 and 4 according to the modified method previously described (Lee *et al*. 1987). Grade 1 means no angiogenesis, while grade 4 indicates the most pronounced angiogenesis.

All surgical procedures were performed under pentobarbital anesthesia (Dainabot, Osaka, Japan). The animal ethics in all animal experiments met the standards required by the UKCCR Guidelines for the Welfare of Animals on Experimental Neoplasia (second edition), as stated in the ‘Instructions to Authors’ forms.

### Statistical analysis

Results are presented as mean±s.e.m. Statistical comparisons were performed using the paired Student's *t*-test. DAS analysis was performed according to the Mann–Whitney *U*-test. A probability value of *P*<0.05 was considered significant.

## Results

### Effect of KIN-841 on angiogenesis in CAM

**Figure 1:**
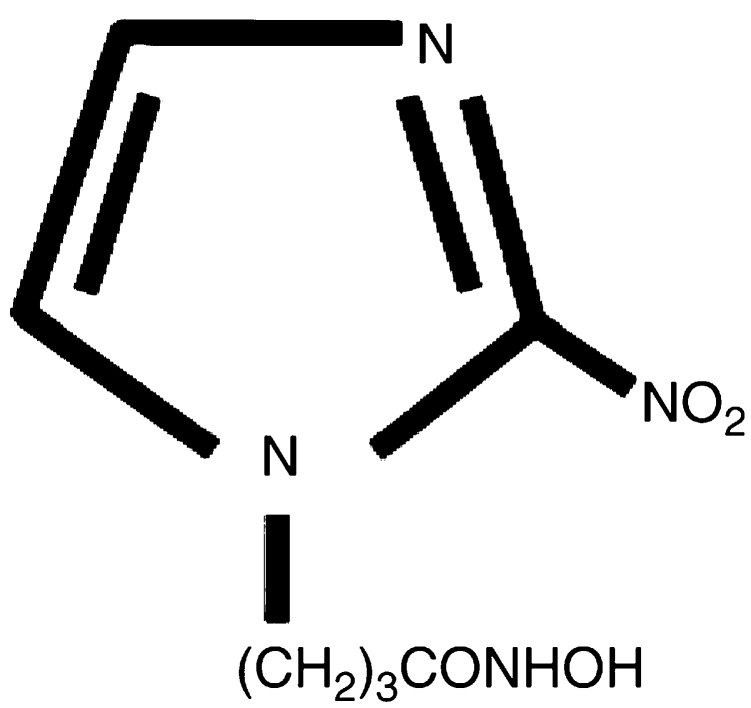
Structure of KIN-841.

The antiangiogenic activities of ten 2-nitroimidazole derivatives (including seven KIN compounds (KIN-801, KIN-804, KIN-806, KIN-808, KIN-811, KIN-831 and KIN-841), two known 2-nitroimidazole derivatives (misonidazole and etanidazole) and 2-nitroimidazole) were determined in the presence of 200 *μ*g CAM^−1^ using CAM assay under normoxic condition. KIN-808 and KIN-841 were found to inhibit angiogenesis (52.3±2.9 and 75.3±1.7%, respectively). On the other hand, the inhibitory activities of misonidazole and etanidazole were low (17.8±3.8 and 38.7±1.9%, respectively). Among the compounds tested, the most potent inhibitor was KIN-841 with a CH_2_(CH_2_)_2_CONHOH moiety at the 1-position of 2-nitroimidazole in the molecule (Figure 1

and Figure 2A

**Figure 2:**
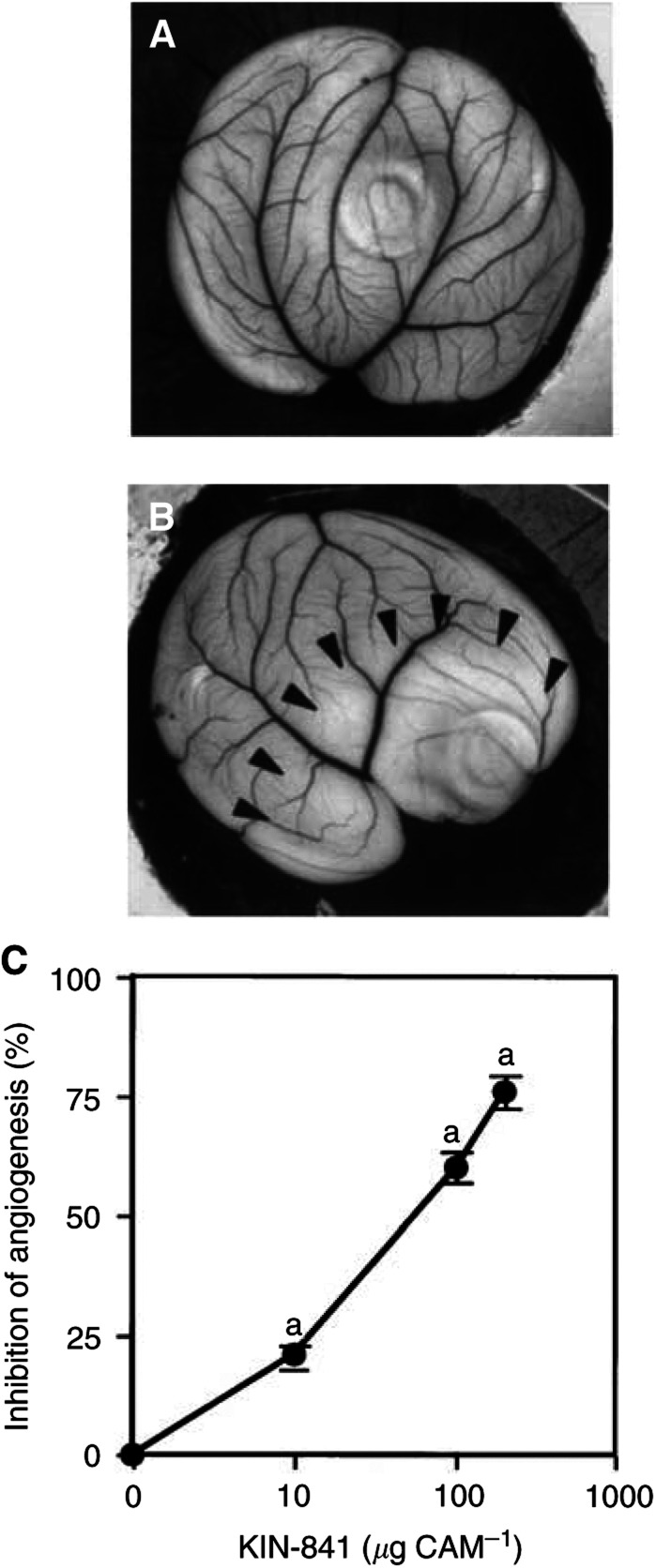
Inhibitory effect of KIN-841 on *in vivo* angiogenesis in CAM. No (**A**) or 100 *μ*g ml^−1^ (**B**) KIN-841 was added to the CAM surfaces of 4-day-old fertilized eggs, and then the eggs were incubated for 48 h as described in Materials and Methods. KIN-841 produced an avascular zone (surrounded by arrows), indicating angiogenic inhibition. (**C**) Dose-dependent inhibition of *in vivo* angiogenesis in CAM by KIN-841. ^a^*P*<0.01 *vs* controls.

,B). KIN-841 suppressed the *in* *vivo* angiogenesis dose-dependently with an ED_50_ of 50 *μ*g CAM^−1^ (Figure 2C).

### Effect of KIN-841 on proliferation of endothelial cells and tumour cells under normoxia

**Figure 3:**
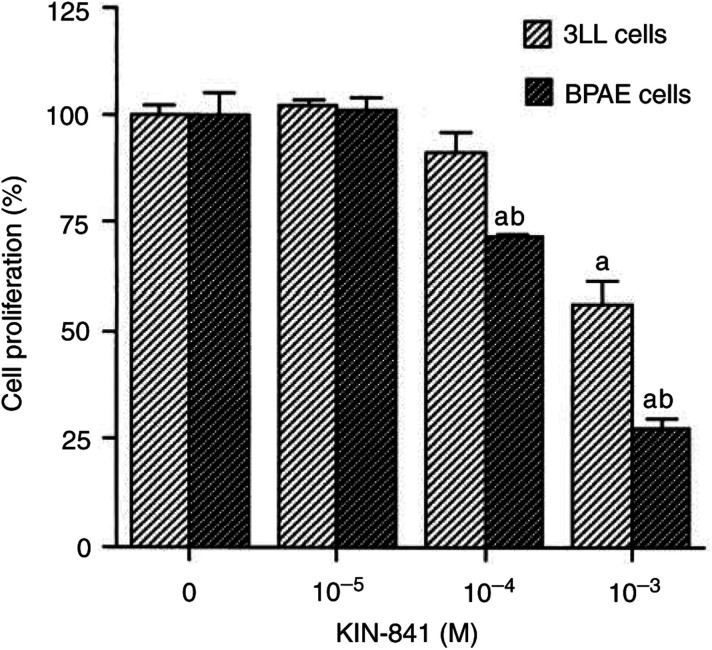
Inhibitory effect of KIN-841 on proliferation of BPAE cells and 3LL cells under normoxia. Cells were treated for 48 h with KIN-841 at the various concentrations indicated. ^a^*P*<0.05 *vs* controls; ^b^*P*<0.05 *vs* normoxia.

BPAE cells and 3LL cells were incubated with various concentrations of KIN-841 in the proliferation assay (Figure 3

). KIN-841 showed a direct inhibitory effect on the proliferation of BPAE cells at 10^−4^ M (*P*<0.05). On the other hand, the proliferation of 3LL cells was not inhibited by 10^−4^ M KIN-841. KIN-841 showed a more potent inhibition of the proliferation against the endothelial cells than of tumour cells (*P*<0.05).

### Effect of KIN-841 on proliferation of tumour cells under hypoxia

**Figure 4:**
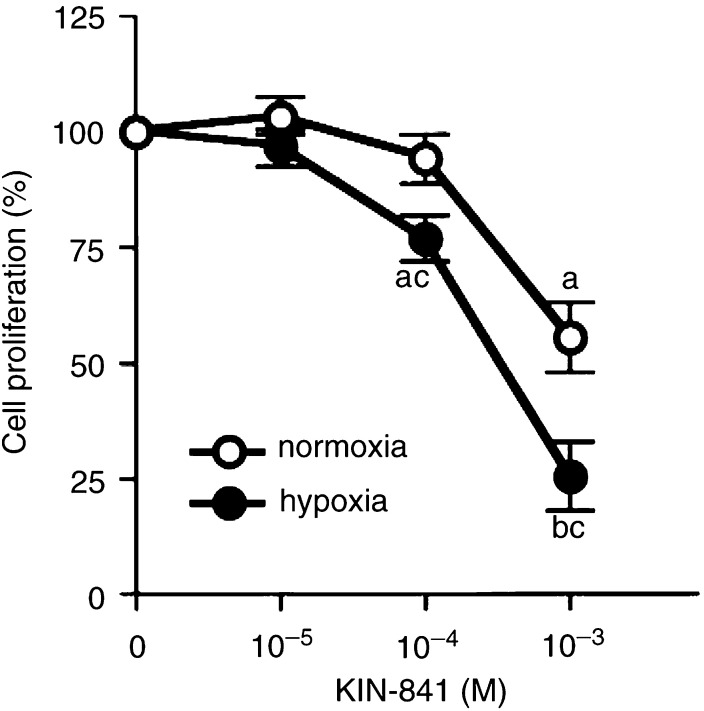
Inhibitory effect of KIN-841 on proliferation of 3LL cells under hypoxia. Cells were treated for 48 h with KIN-841 at the various concentrations indicated under normoxia or hypoxia. ^a^*P*<0.05 *vs* controls; ^b^*P*<0.01 *vs* controls; ^c^*P*<0.05 *vs* normoxia.

Previously, all the compounds tested other than 2-nitroimidazole in CAM assay under normoxia were reported to exhibit similar high levels of radiosensitising activity against hypoxic tumour cells (Nagasawa *et al*. 1992). The radiosensitising activity was shown to correlate with specific cytotoxicity in hypoxic tumour cells. Therefore, subsequent experiments under hypoxic condition were carried out with the most potent angiogenic inhibitor under normoxia, KIN-841. 3LL cells were cultured with or without KIN-841 in culture chambers under hypoxia or normoxia for 48 h (Figure 4

). Under the hypoxic conditions, the cell growth was slow and KIN-841 inhibited the proliferation of 3LL cells at 10^−4^–10^−3^ M (*P*<0.01). On the other hand, this inhibitory activity was weak when the cells were cultured under normoxic conditions (*P*<0.05 *vs* hypoxia). The inhibition of KIN-841 on 3LL cell proliferation was about five times more potent under hypoxia than under normoxia.

### Effect of KIN-841 on production of angiogenic factor by hypoxic tumour cells

**Figure 5:**
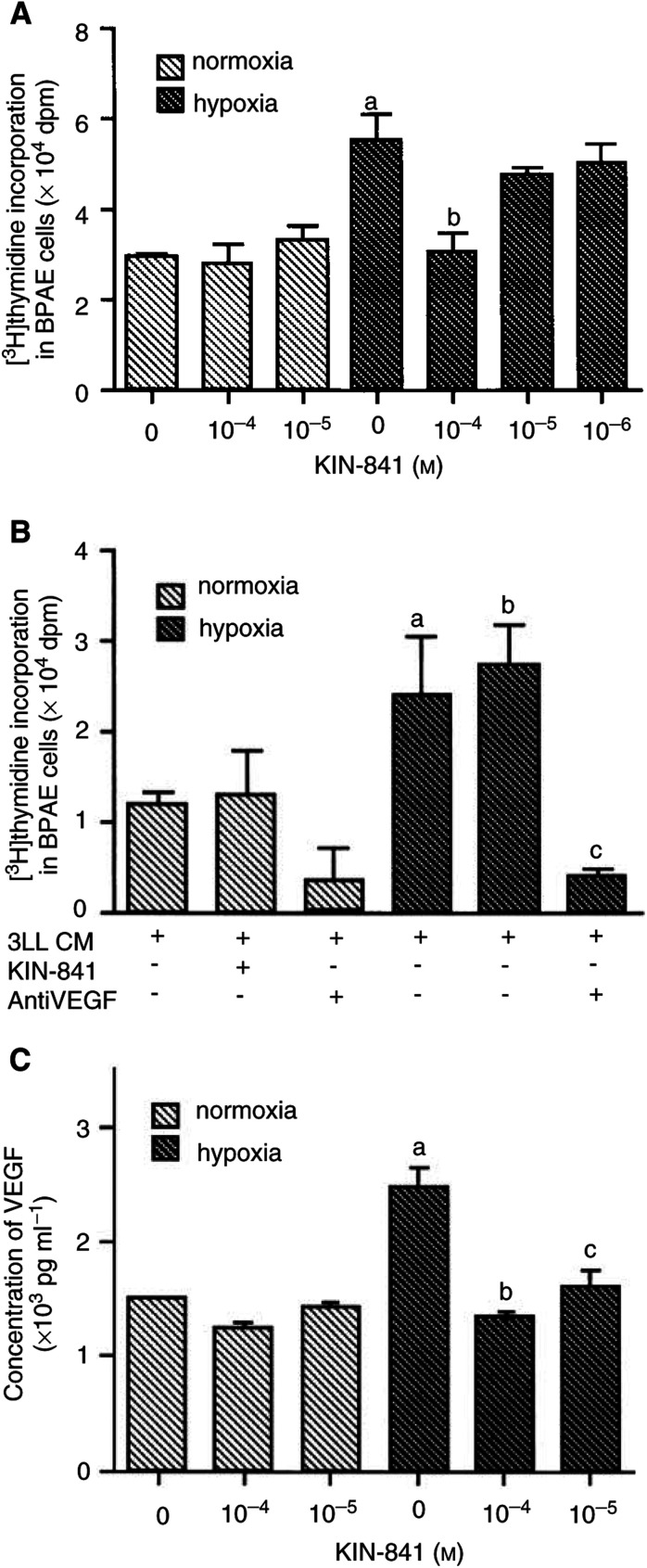
Inhibitory effect of KIN-841 on the production of angiogenic factor in 3LL cells under hypoxia. (**A**) Effect of 3LL cell-CM treated with KIN-841 at the various concentrations indicated under normoxia or hypoxia on [^3^H]thymidine incorporation in BPAE cells. ^a^*P*<0.01 *vs* normoxia controls; ^b^*P*<0.01 *vs* hypoxia controls. (**B**) Effects of KIN-841 and anti-VEGF antibody on stimulation of [^3^H]thymidine incorporation in BPAE cells by 3LL cell-CM incubated under normoxia or hypoxia. ^a^*P*<0.05 *vs* normoxia controls; ^b^*P*<0.05 *vs* normoxia controls; ^c^*P*<0.01 *vs* hypoxia controls. (**C**) VEGF concentration in 3LL cell-CM treated with KIN-841 at the various concentrations indicated under normoxia or hypoxia. ^a^*P*<0.05 *vs* normoxia controls; ^b^*P*<0.05 *vs* hypoxia controls; ^c^*P*<0.01 *vs* hypoxia controls.

It is well known that hypoxic tumour cells produce significant amounts of many kinds of factors that initiate angiogenesis. To determine whether KIN-841 suppresses the production of angiogenic factors by 3LL cells under hypoxia, the serum-free CM was obtained from 3LL cells that were treated with various concentrations of KIN-841 under normoxia or hypoxia. The levels of the angiogenic factors in the CM were measured by [^3^H]thymidine incorporation into BPAE cells (Figures 5A and B

) and by ELISA (Figure 5C). The CM from 3LL cells cultured under hypoxia significantly stimulated thymidine uptake into BPAE cells compared with that obtained from the cells cultured under normoxia (*P*>0.05, Figure 5A). This activity in CM of hypoxic tumour cells was 1.8 times higher than that in normoxic cells. The stimulating activity was decreased when the tumour cells were incubated with KIN-841 under hypoxia (*P*>0.01, Figure 5A). In contrast, the direct addition of KIN-841 did not block the thymidine uptake induced by the CM from 3LL cells (Figure 5B). However, the anti-mouse VEGF antibody potently blocked the stimulating activity of the CM from hypoxic tumour cells, suggesting that the angiogenic factors in the CM are VEGFs (Figure 5B). Figure 5C shows the levels of VEGF in the CM of 3LL cells treated with various concentrations of KIN-841 under the same conditions as in Figure 5A. In 3LL cells, VEGF production was significantly induced by hypoxia (*P*<0.01), and KIN-841 at 10^−5^–10^−4^ M substantially inhibited VEGF production (*P*<0.05).

### Effect of KIN-841 on tumour-induced angiogenesis

**Figure 6:**
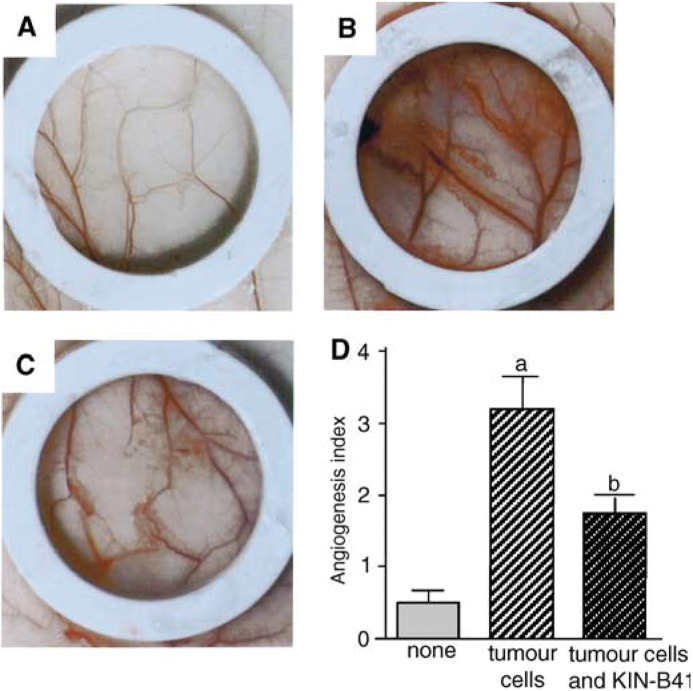
Inhibition of 3LL-cell-induced angiogenesis by KIN-841 as determined using the mouse dorsal air sac method. Dorsal skin of the mice 7 days after implantation of a chamber filled with PBS alone (**A**) or 3LL cells (**B** and **C**). The implanted mice were treated with 200 mg kg^−1^ day^−1^ of KIN-841 for 4 days between days 1 and 6, intraperitoneally (**C**). In (**D**), the grade of angiogenesis induced by 3LL cells is shown using the indexes described in Materials and Methods. ^a^*P*<0.01 tumour-free controls; ^b^*P*<0.05 *vs* tumour controls.

To determine whether KIN-841 might have therapeutic value *in vivo* in the treatment of tumour-induced angiogenesis, the effect of KIN-841 on 3LL-cell-induced angiogenesis was investigated using the mouse dorsal air sac method (Figure 6

). In mice implanted with a Millipore chamber containing saline, angiogenesis hardly occurred (group A, Figure 6A). On the other hand, in the mice of group B, implanted with a chamber containing 3LL cells and then treated with saline, potent angiogenesis was induced (Figure 6B). When the mice were intraperitoneally administered with 200 mg kg^−1^ day^−1^ of KIN-841 (group C, Figure 6C), the angio-cgenesis was significantly suppressed. The angiogenesis indexes of group B (3LL cells alone) and group C (3LL cells and KIN-841) were 3.2±0.44 and 1.75±0.20, respectively (Figure 6D). KIN-841 significantly inhibited *in vivo* angiogenesis induced by 3LL cells in the dorsal air sac assay (*P*<0.05).

## Discussion

Tumour angiogenesis is a complex multistep process involving the following: (1) production of angiogenic factors by tumour cells, especially hypoxic tumour cells; (2) upregulation of the biological functions of vascular endothelial cells by angiogenic factors; (3) degradation of the basement membrane of endothelial cells by enzymes; (4) migration of endothelial cells; (5) proliferation of endothelial cells; and (6) tube formation of endothelial cells. Interruptions in certain steps of this series of biochemical events may prevent angiogenesis. In particular, the inhibition of the early steps of the process may promote antiangiogenesis. The prevention of the first step of angiogenesis that involves the production of angiogenic factors by hypoxic tumour cells is potently effective in promoting tumour-specific antiangiogenesis.

There are many factors implicated in angiogenesis. The production of several angiogenic factors, including VEGF, bFGF and PDGF (Minchenko *et al*. 1994; Gleadle *et al*. 1995; Kuwabara *et al*. 1995), is upregulated by hypoxia. In solid tumours, a more rapid increase in the number of oxygen-consuming tumour cells than can be sustained by the oxygen supply to the cells from the microvasculature occurs, resulting in tissue hypoxia. Continuous hypoxia results in the expression of the genes coding for angiogenic factors, which are produced in secretory forms. Angiogenic factors act on their receptors on endothelial cells in an autocrine and paracrine manner, thereby causing the activation of endothelial cells, which leads to angiogenesis and subsequent progressive tumour growth and metastasis. Therefore, the prevention of the production of angiogenic factors in hypoxic tumour cells may result in the potent inhibition of angiogenesis, tumour growth and metastasis.

In this study, we attempted to (1) inhibit the production of angiogenic factors in hypoxic tumour cells using 2-nitroimidazole derivatives, which covalently bind to hypoxic tumour cells and are cytotoxic against hypoxic tumour cells, and (2) simultaneously disrupt the endothelial cell function using the same agent, to achieve potent inhibition of tumour-specific angiogenesis. We found the hypoxia-dependent 2-nitroimidazole KIN-841, with a hydroxamate moiety in the side chain, to be the most potent angiogenic inhibitor by an *in vivo* assay using the CAM under normoxia (Figure 1 and Figure 2).

In order to determine the antiangiogenic mechanism of KIN-841, its effect on the proliferation of endothelial cells was examined. KIN-841 inhibited the proliferation of vascular endothelial cells under normoxia (Figure 3). Its inhibitory effect on endothelial cells was more potent than that on tumour cells. These results suggest that KIN-841 inhibits angiogenesis by disrupting vascular endothelial cell growth. Under hypoxic conditions, KIN-841 inhibited the proliferation of tumour cells more potently than under normoxia (Figure 4). The CM from 3LL cells under hypoxia could stimulate [^3^H]thymidine incorporation into vascular endothelial cells. This stimulating activity in CM was estimated to be because of the presence of VEGFs (Figure 5B, C). In contrast, the CM of KIN-841-treated hypoxic tumour cells decreased the stimulating activity and VEGF level (Figure 5A, C). KIN-841 did not directly inhibit the stimulating activity (Figure 5B). These results indicated that KIN-841 can suppress the production of angiogenic factors, VEGFs, derived from hypoxic tumour cells and may inhibit tumour-specific angiogenesis.

There is no question regarding the importance of VEGFs in angiogenesis and expansion of solid tumours, because VEGF particularly affects vascular endothelial cells and, consequently, stimulates all steps of angiogenesis. However, several reports revealed that angiogenic factors other than VEGF play important roles in angiogenesis (Hartmann *et al*. 1999; Koga *et al*. 2001; Shimo *et al*. 2001). In melanoma cell lines, a strong correlation between VEGF expression and tumorigenesis was not observed, although VEGF production was induced under the hypoxic condition (Hartmann *et al*. 1999). Angiogenin production was reported to be upregulated in melanoma cell lines but not in normal melanocytes under hypoxic condition. The level of induction of angiogenin correlated with the metastatic potential of the cell lines. Furthermore, a strong expression of angiogenin was observed in melanomas and metastases from patients, but was not observed in benign nevi. These indicated that angiogenin is the most potent angiogenic factor in melanomas. Various approaches to inhibiting angiogenic factor signalling pathways have been adopted using specific antibodies against various angiogenic factors. A monoclonal antibody against VEGF inhibited angio-genesis and prevented tumour growth in solid tumours (Borgstrom *et al*. 1996; Brekken and Thorpe, 2001). However, a specific antibody against one angiogenic factor cannot completely inhibit angiogenesis, which depends on two or more factors or a different type of angiogenic factor. This problem may be overcome if hypoxia-induced angiogenic factor-producing cells can be attacked and the production of angiogenic factors by hypoxic cells can be inhibited. KIN-841 may be able to inhibit the production of all types of hypoxia-induced angiogenic factors in tumour cells.

We further determined the effect of KIN-841 on tumour-induced angiogenesis using the mouse dorsal air sac method. KIN-841 exhibited inhibitory activity on the angiogenic response triggered by mouse malignant 3LL cells (Figure 6). This result suggested that KIN-841 inhibits tumour growth and metastasis *in vivo* by disrupting angiogenesis. Recently, the oxygen tensions in a neck node of a squamous cell carcinoma and adjacent normal tissue were measured (Adam *et al*. 1999). Normal tissue showed a typical Gaussian distribution of oxygen tensions with a median value between 40 and 60 mmHg. On the other hand, tumours showed a distribution of much lower oxygen tension with a median value of less than 10 mmHg. Since the oxygen tension in normal tissue is slightly low, KIN-841 may express more potent *in vivo* antiangiogenesis because of its high affinity to hypoxic tumour cells and slightly hypoxic endothelial cells.

In conclusion, potent inhibition of tumour-specific angiogenesis can be induced by simultaneously preventing the production of angiogenic factors by hypoxic tumour cells and vascular endothelial cell function involved in cell proliferation under normoxia in the presence of a novel hypoxia-dependent 2-nitroimidazole derivative, KIN-841. Furthermore, the hypoxia-dependent KIN-841 may be able to target effectively tumour-associated vasculatures and exhibit potent antiangiogenic activity in solid tumours and prevent tumour metastasis.
